# Prevalence and associated factors of prenatal depression in pregnant Korean women during the COVID-19 pandemic: a cross-sectional study

**DOI:** 10.4069/kjwhn.2023.11.22

**Published:** 2023-12-28

**Authors:** Mi-Eun Kim, Ha-Neul Jung

**Affiliations:** Department of Nursing Science, Jeonju University, Jeonju, Korea

**Keywords:** COVID-19, Depression, Pregnancy, Prenatal education

## Abstract

**Purpose:**

This study investigated the effects of prenatal education characteristics, pandemic-related pregnancy stress, and health behaviors during pregnancy on prenatal depression in pregnant women during the coronavirus disease 2019 (COVID-19) pandemic.

**Methods:**

The participants were 180 pregnant Korean women, recruited from internet communities for pregnancy preparation, childbirth, and childcare, from July 5 to 15, 2022. The collected data were analyzed using the t-test, analysis of variance, the Mann-Whitney U-test, the Kruskal-Wallis test, and multiple regression analysis.

**Results:**

The scores for pandemic-related pregnancy stress (24.50±6.37) and health behaviors during pregnancy (67.07±9.20) were high. Nearly half of the participants (n=89, 49.4%) presented with prenatal depression, with scores of 10 or greater. Prenatal depression had a positive correlation with gestational age (r=.18, *p*=.019) and pandemic-related pregnancy stress (r=.27, *p*<.001), and a negative correlation with health behaviors during pregnancy (r=–.42, *p*<.001). The factors associated with prenatal depression were pandemic-related pregnancy stress (t=4.70, *p*<.001), marital satisfaction (dissatisfied) (t=3.66, *p*<.001), pregnancy healthcare practice behaviors (t=–3.31, *p*=.001), family type (weekend couple) (t=2.84, *p*=.005), and gestational age (t=2.32, *p*=.022). The explanatory power of these variables was 38.2%.

**Conclusion:**

Since participants had a high level of prenatal depression during the pandemic, and infectious diseases such as COVID-19 may recur, strategies should be developed to improve pregnant women’s mental health with consideration of the unique variables that are relevant in a pandemic. It is also necessary to develop efficient online prenatal education programs that can be implemented even in special circumstances such as social distancing, and to evaluate their effectiveness.

## Introduction

During pregnancy, women undergo unique physiological, psychological, and social changes. They are exposed to a considerable amount of visible and potential stress, which can make them more susceptible to depression and anxiety [1]. Before the outbreak of the coronavirus disease 2019 (COVID-19) prenatal stress, encompassing general worries and fears about pregnancy, was reported as being more prevalent among pregnant women with a history of mental illness, those who were younger, or those with lower incomes and educational levels [2]. However, during the COVID-19 pandemic, even pregnant women without these risk factors appeared to have experienced high levels of prenatal stress, and some exhibited symptoms of dissociative disorder and posttraumatic stress disorder (PTSD) [2] . There was also a significant increase in depression and anxiety disorders during the pandemic, which indicates that the psychological impact of the pandemic appeared to have been a threat to all pregnant women, not just those with vulnerable characteristics [2]. The decline in mental health among women during pregnancy can be attributed to several factors, such as the unknown effects of COVID-19 on the health of both the mother and fetus, changes and restrictions in prenatal checkup routines and service facilities, and isolation from social support networks due to social distancing measures [3].

Recent research suggests that increased stress during pregnancy and prenatal depression, particularly during a pandemic, can disrupt the maternal-fetal bond [4]. This heightened stress can trigger an overactive response in the fetus, leading to an excessive release of stress hormones in the mother. This, in turn, activates the immune system, potentially causing issues with inflammation and immune regulation [5]. Consequently, this can result in adverse birth outcomes, such as preterm birth or the delivery of babies that are small or of low birthweight for their gestational age [3].

During the pandemic, pregnant women reported significantly elevated stress levels compared to the period before. Their depression was intensified by the fear of infection and a lack of adequate support during childbirth [6]. Prior research has identified marital satisfaction as a significant determinant of prenatal depression [7]. Lower marital satisfaction has been linked to heightened prenatal depression and a reduction in healthcare practices during pregnancy [8]. Prenatal depression is also affected by gestational age, with physical and mental stress escalating in the third trimester. This demonstrates the need for meticulous management of prenatal depression, taking into account the gestational week.

Improving behaviors related to pregnancy healthcare can serve as an effective strategy for reducing prenatal depression. The frequency of breakfast consumption, sleep duration, and drinking habits all have an impact on prenatal depression; thus, a diet rich in vitamins and increased physical activity are recommended during pregnancy [9]. Even amidst a pandemic, participating in online fitness classes can increase physical activity levels, thereby bolstering pregnant women’s resilience against prenatal depression. Moreover, factors associated with physical activity and sleep during pregnancy play a significant role in managing pandemic-related stress, underscoring the importance of reinforcing healthcare practices among pregnant women [10].

Prenatal education can improve the healthcare practices of pregnant women, instilling a sense of preparedness for pregnancy and parenthood. This not only boosts their confidence in childbirth but also mitigates prenatal depression and wards off postpartum depression [11-13]. Prenatal education must evolve to meet the times and the specific needs of pregnant women. However, traditional approaches often overlook the individual circumstances of pregnant women and tend to generalize their experiences [14].

COVID-19 has been linked to significant changes in the mental health, quality of life, attitudes, and lifestyle of pregnant women. There has been a notable increase in stress and depression during this period. Social distancing measures have curtailed their physical activities during leisure time, leading to an increase in time spent at home. Furthermore, there has been a heightened interest in the complications, epidemiology, and treatment of infectious diseases such as COVID-19 [15]. Consequently, this study aims to assess the current state of prenatal education and the impact of pandemic-related pregnancy stress and healthcare practices on prenatal depression. The goal is to provide foundational data and evidence to develop intervention strategies for prenatal education that can enhance mental health and healthcare practices among pregnant women.

The specific aims of this study were as follows:

1) To examine the general and obstetric characteristics of pregnant women and their prenatal education during the COVID-19 pandemic

2) To assess the levels of pandemic-related pregnancy stress, healthcare practices, and prenatal depression during the COVID-19 pandemic

3) To analyze the differences in these factors among pregnant women based on their general, obstetric, and prenatal education characteristics

4) To investigate the correlation between these characteristics, pandemic-related stress, healthcare practices, and prenatal depression

5) To determine the factors associated with prenatal depression among pregnant women during the COVID-19 pandemic

## Methods

**Ethics statement:** This study was approved by the Institutional Review Board of Jeonju University (No. jjIRB-220728-HR-2022-0511). Obtaining written informed consent was exempted due to the complete anonymity inherent to the design of the online survey. The study was conducted in line with the Declaration of Helsinki.

### Research design

This cross-sectional, correlational study aimed to investigate the impact of prenatal education characteristics, pandemic-related pregnancy stress, and pregnancy healthcare practice behaviors on prenatal depression during the COVID-19 pandemic. This study adhered to the STROBE (Strengthening the Reporting of Observational Studies in Epidemiology) guidelines (https://www.strobe-statement.org).

### Participants

Women aged 18 years or older, who were pregnant during the COVID-19 pandemic were recruited via online community posts through convenience sampling. The third trimester (28 weeks or more) was selected for participant selection, based on prior research [16] that indicated a higher prevalence of prenatal depression during this stage of pregnancy. The decision to concentrate on this group was further substantiated by the results of the Ministry of Health and Welfare's National Mental Health Survey for the second quarter of 2022. This survey showed a significant surge in depression rates during the COVID-19 outbreak in December 2021, with rates ranging from 18.1 to 22.8%, representing a more than fivefold increase from 2019 [17]. Therefore, it was deemed necessary to investigate depression in pregnant women during this critical period. Women who had difficulty with Korean language literacy or were under 28 weeks of pregnancy were excluded. The sample size was determined using the G*power 3.1.9.2 program, with a significance level of 0.05, a medium effect size of 0.15, a power of 0.85, and 15 predictors related to prenatal depression, general, obstetric, and prenatal education characteristics and the minimum sample size was determined to be 153 participants. The target was set at 180 participants to account for a potential 15% dropout rate [18]. As there were no incomplete or insufficient responses, the final analysis was conducted with a sample size of 180 (100%).

### Instruments

#### Prenatal depression

The investigators obtained permission to utilize the Korean version [19] of the Edinburgh Postnatal Depression Scale (EPDS) [20]. Although the EPDS tool was initially created to evaluate postnatal depression, it has been validated for use in measuring depression during pregnancy [21]. The tool is comprised of 10 items, each rated on a 4-point Likert scale. It evaluates symptoms such as depression, anxiety, and suicidal ideation experienced in the past week. Responses range from "not at all" (0 points) to "very much" (3 points). With the exception of items 1, 2, and 4, all items are scored in reverse. Higher scores (possible range: 0-30) indicate more severe prenatal depression and the cutoff for depression in Korean women was scores of 10 or higher [21]. The reliability was confirmed by a Cronbach's α value of .85 in a prior study with Korean women [19] and .86 in the present study.

#### Pandemic-related pregnancy stress

The Pandemic-related Pregnancy Stress Scale (PREPS) utilized in this study was adapted from the scale originally developed by Preis et al. [22] and subsequently translated into Korean by Kim and Heo [23], with the developer's permission. The scale comprises two subdomains: “perinatal infection stress (3 items)” and “preparedness stress (4 items).” Each item is rated on a 5-point scale (1 not at all, to 5 very much) and higher scores (possible range: 7-35) indicate greater pandemic-related pregnancy stress. Cronbach's α values for the original scale ranged from .68 to .86 [22]. The overall reliability of the seven items in the Korean version Cronbach's α of .87, with subdomain values ranging from .81 to .85 [23]. Cronbach's α was .92 for this study, with subdomain values between .85 and .91.

#### Pregnancy healthcare practice behavior

The 20-item Prenatal Healthcare Behavior Scale, originally developed by Wang et al. [24]and later revised and supplemented by Wang and Kim [25], was adapted with the developers' permission. The adapted version comprises 17 items in the following six subdomains: medication management (3 items), physical care/hygiene (4 items), prenatal care/education (2 items), activity/rest (3 items), nutrition management (3 items), and mental health (2 items). Each item is scored on a 5-point scale (1 not at all, to 5 very well) and higher scores (possible range: 17-85) indicate a higher level of pregnancy healthcare practice behavior. In previous research [25], Cronbach's α was .72, while in the current study Cronbach's α was .83.

#### General, obstetric, and prenatal education characteristics

General characteristics encompassed age, marital status, family structure, marital satisfaction, job loss, and income changes resulting from the COVID-19 pandemic. Obstetric characteristics included gestational age, prenatal checkups, parity, planned pregnancy, smoking and drinking habits during pregnancy, method of conception, high-risk pregnancy status, preferred sex of the fetus, desired childbirth method, alterations in childbirth plans due to the COVID-19 pandemic, self-quarantine experiences during pregnancy, changes in prenatal checkups, and COVID-19 diagnoses during pregnancy. Finally, prenatal education characteristics centered on the need for and interest in prenatal education, methods of acquiring prenatal information during pregnancy, preferred prenatal education methods and modes, changes in prenatal education participation due to the COVID-19 pandemic (including reasons for these changes), and the receipt of prenatal education during the pandemic. This last category also included reasons for not receiving education (if applicable), satisfaction with the received prenatal education, the number of prenatal education classes attended, and whether the participant was accompanied by a husband or guardian, if any.

### Data collection

The data were collected via an online survey from July 5 to 15, 2022. The survey was disseminated through internet communities in Korea focused on pregnancy preparation, childbirth, and childcare. Emails were dispatched to the coordinators of these online communities, soliciting their help in data collection. An announcement about the survey, along with a link to the online questionnaire, was posted on the community boards. Access was granted only to those who agreed to participate in the study. Both the community boards and the online questionnaire clearly stated that participants could withdraw from the study at any time. Agreement to participate voluntarily, after reading the relevant information in the online questionnaire, was a prerequisite for participation in the study. The survey took approximately 15 minutes to complete. As a token of appreciation, participants who completed the survey received a mobile beverage voucher (worth approximately 5 US dollars) via text message within 14 days of completion.

### Data analysis

SPSS for Windows ver. 27.0 (IBM Corp., Armonk, NY, USA) was used to analyze the data. Pregnant women’s general and obstetric characteristics, prenatal education characteristics, pandemic-related pregnancy stress, pregnancy healthcare practice behaviors, and prenatal depression during the COVID-19 pandemic were analyzed using frequency analysis and descriptive statistics. The differences in these factors among pregnant women based on their general, obstetric, and prenatal education characteristics were analyzed using the t-test, analysis of variance, the Mann-Whitney U-test, and the Kruskal-Wallis test, with post-hoc tests conducted using the Scheffé test. The correlations among these characteristics, pandemic-related stress, healthcare practices, and prenatal depression among pregnant women were analyzed using Pearson correlation coefficients. The factors that influenced prenatal depression among pregnant women during the COVID-19 pandemic were identified using multiple regression analysis.

## Results

### General, obstetric, and prenatal education characteristics of participants

Table 1 presents the general, obstetric, and prenatal education characteristics of the 180 participants. The average age of the participants was 32.22±2.86 years. A significant majority, 156 participants (86.7%), reported no job loss, while 127 participants (70.6%) indicated no change in their income due to the COVID-19 pandemic.

The median gestational age of the participants was 31 weeks and 6 days. Most participants (n=156, 86.7%) did not alter their childbirth plans due to the pandemic and 119 participants (66.1%) did not undergo self-quarantine during their pregnancy. Furthermore, 134 participants (74.4%) reported no changes to their prenatal checkups, and 153 participants (85.0%) were not diagnosed with COVID-19 during their pregnancy.

The most popular method of obtaining prenatal information was through the internet (blogs, online communities, YouTube), reported by 152 participants (84.4%) through multiple responses. Knowledge transfer and practice education were identified as the most preferred types of prenatal education by 140 participants (77.8%). Of the 46 participants (25.6%) who experienced changes in their prenatal education due to the pandemic, 29 (63%) stated that these changes were due to schedule alterations or cancellations by educational institutions, based on multiple responses. Among all participants, 96 (53.3%) received prenatal education during this period. Of the remaining 84 participants (46.7%) who did not receive education, the most frequently cited reason was “restrictions on gatherings due to social distancing,” reported by 45 participants (53.6%) through multiple responses.

### Pandemic-related pregnancy stress, pregnancy healthcare practice behavior, and prenatal depression

The participants’ pandemic-related pregnancy stress was 24.50±6.37 (3.50±0.91 out of 5 points), which indicates a high level of stress. The mean score for preparedness stress (14.22±3.88; point average, 2.03±0.55) was slightly higher than for perinatal infection (10.29±3.09; point average, 1.47±0.40) (Table 2).

The overall mean score for pregnancy healthcare practice behavior was also high, at 67.07±9.20 (3.95±0.54 out of 5 points). The average score for prenatal depression was 8.85±5.31 and 91 participants (50.6%) fell within the normal range (0-9, mean, 4.51±3.12) whereas 89 participants (49.4%) were categorized as having depression (score of 10 or greater; mean, 13.28±2.86) (Table 2).

### Differences in pandemic-related pregnancy stress, pregnancy healthcare practice behavior, and prenatal depression according to the participants’ characteristics

The method of pregnancy (natural or not; Z=–2.01, *p*=.045) and changes in the childbirth plan due to the COVID-19 pandemic (Z=–3.62, *p*<.001) were identified as statistically significant factors influencing pandemic-related pregnancy stress, based on the obstetric characteristics. Regarding prenatal education characteristics, the need for prenatal education (χ^2^=22.51, *p*<.001), the preferred method of prenatal education (χ^2^=9.41, *p*=.009), modifications in prenatal education participation due to the COVID-19 pandemic (F=5.54, *p*=.005), and receiving prenatal education during the COVID-19 pandemic (t=3.49, *p*=.001) were also found to have statistically significant associations with pandemic-related pregnancy stress. Post-hoc analysis revealed that those who experienced changes in prenatal education due to the COVID-19 pandemic exhibited higher levels of pandemic-related pregnancy stress than those who did not (Table 3).

Based on the general and obstetric characteristics of the participants, several factors were found to significantly influence pregnancy healthcare practice behaviors. These factors include marital status (Z=–3.07, *p*=.002), family type (χ^2^=7.17, *p*=.028), marital satisfaction (χ^2^=18.94, *p*<.001), and changes in income due to the COVID-19 pandemic (t=2.13, *p*=.035). Other influential factors included prenatal checkup (Z=–4.28, *p*<.001), planned pregnancy (t=2.94, *p*=.004), smoking during pregnancy (Z=–2.62, *p*=.009), drinking during pregnancy (Z=–3.54, *p*<.001), high-risk pregnancy (Z=–2.46, *p*=.014), and experience of self-quarantine during pregnancy (t=–2.59, *p*=.010). In terms of prenatal education characteristics, the desired method of prenatal education (χ^2^=16.38, *p*<.001), the preferred mode of prenatal education (χ^2^=13.21, *p*=.001), changes in prenatal education participation due to the COVID-19 pandemic (F=3.41, *p*=.035), and receiving prenatal education during the COVID-19 pandemic (t=2.67, *p*=.008) were also found to have a significant impact on pregnancy healthcare practice behaviors. Post-hoc analysis revealed that participants who did not experience changes in prenatal education demonstrated higher levels of pregnancy healthcare practice behaviors than those who did (Table 3).

Statistical analysis revealed the following characteristics that were likely to significantly impact prenatal depression: family type (χ^2^=11.84, *p*=.003), marital satisfaction (χ^2^=11.79, *p*=.003), job loss due to the COVID-19 pandemic (Z=–2.21, *p*=.027), and changes in income as a result of the pandemic (t=–3.35, *p*=.001). Other influential factors were prenatal checkups (Z=–2.73, *p*=.006), whether the pregnancy was planned (t=–2.25, *p*=.026), alcohol consumption during pregnancy (Z=–2.48, *p*=.013), the desired sex of the fetus (t=–2.54, *p*=.012), and the preferred method of childbirth (χ^2^=6.37, *p*=.041). The experience of self-quarantine during pregnancy (t=3.26, *p*=.001) and changes in prenatal checkups due to the pandemic (t=2.27, *p*=.024) also showed a high likelihood of affecting prenatal depression. In terms of prenatal education characteristics, the preferred mode of prenatal education (χ^2^=6.79, *p*=.034) had a statistically significant association with prenatal depression (Table 3).

### Correlations among the participants’ characteristics and main variables

Prenatal depression in pregnant women showed a slight positive correlation with both gestational age (r=.18, *p*=.019) and stress related to the pandemic (r=.27, *p*<.001). Conversely, it demonstrated a moderately strong negative correlation with behaviors related to pregnancy healthcare practices (r=–.42, *p*<.001) (Table 4).

### Factors associated with prenatal depression during the COVID-19 pandemic

To identify factors influencing prenatal depression, we dummy-coded general and obstetric characteristics that demonstrated significant impacts. These included family type, marital satisfaction, job loss, changes in income, prenatal checkups, planned pregnancy, alcohol consumption during pregnancy, desired sex of the fetus, preferred childbirth method, self-quarantine experience during pregnancy, and changes in prenatal checkups. Additionally, the preferred mode of prenatal education, which fell under prenatal education characteristics, was also included. Alongside these, we included gestational age and key variables such as pandemic-related pregnancy stress and pregnancy healthcare practice behaviors, both of which showed a correlation with prenatal depression. As a result, a total of 15 independent variables were analyzed in the multiple regression analysis using the enter method. Moreover, the dummy variable for the preferred childbirth method (cesarean section) demonstrated multicollinearity with a tolerance limit value of 0.06 and a variance inflation factor (VIF) value of 18.04. This was also the case for the variable for the preferred childbirth method (natural delivery), which had a tolerance limit value of 0.06 and a VIF value of 17.93. Consequently, the dummy variable with the higher VIF value—specifically, the preferred childbirth method (cesarean section)—was removed prior to conducting the multiple regression analysis (Table 5).

The tolerance limit values for the independent variables fell within a range of 0.52 to 0.91, while the VIF values varied between 1.10 and 1.91. This suggests that multicollinearity was not an issue. The Durbin-Watson statistic registered at 1.81, nearing the standard of 2, but not approaching 0 or 4, thereby affirming the independence of errors. The resulting multiple regression model proved to be significant (F=7.14, *p*<.001), with the 15 independent variables accounting for 38.2% of the variance.

Multiple regression analysis indicated that pandemic-related pregnancy stress (t=4.70, *p*<.001), marital dissatisfaction (t=3.66, *p*<.001), pregnancy healthcare practice behavior (t=–3.31, *p*=.001), being part of a weekend couple (t=2.84, *p*=.005), and advanced gestational age (t=2.32, *p*=.022) were all significant predictors of prenatal depression. This suggests that pregnant women who experienced higher levels of pandemic-related stress, marital dissatisfaction, and lower engagement in pregnancy healthcare practices, those who were part of a weekend couple, and those with a higher gestational age were more likely to experience prenatal depression. During the COVID-19 pandemic, it was found that among the variables influencing prenatal depression, pandemic-related pregnancy stress (β=.29) had the most significant impact.

## Discussion

The present study found that 49.4% of pregnant women experienced prenatal depression, as determined by a score of 10 or higher on the K-EPDS. This rate aligns with a previous study [26] conducted during the COVID-19 pandemic, which reported a high prevalence of 56.3% using the same tool and criteria. This high prevalence sharply contrasts with a 21.1% rate found in a pre-pandemic study [27] in Korea, suggesting a significant increase in prenatal depression during the pandemic. The current study identified various risk factors for prenatal depression, each with a distinct impact. These factors include family type, marital satisfaction, prenatal checkups, planned pregnancy, alcohol consumption during pregnancy, desired sex of the fetus, and preferred childbirth method. These findings align with previous research [28,29] on Korean pregnant women. Additionally, this study incorporated COVID-19-related variables such as job loss, changes in income, self-quarantine experience, and alterations in prenatal checkups, all of which were found to influence prenatal depression. Prior research [30] has underscored that prolonged self-quarantine and disrupted prenatal care due to the pandemic can exacerbate prenatal depression. The severity of depression significantly increased in both pregnant and nonpregnant women when self-quarantine exceeded 50 days. This highlights the need for policy discussions about suitable self-quarantine durations for pregnant women and the importance of monitoring their mental health during repeated outbreaks. Consequently, follow-up studies on prenatal depression based on the duration of self-quarantine are crucial. Moreover, 25.6% of participants experienced changes in their prenatal checkups due to the COVID-19 pandemic, and these women reported higher levels of prenatal depression than those who did not experience changes. However, another study [31] found that 37.1% of pregnant women were unable to receive regular checkups due to the pandemic, a rate higher than that observed in this study. Unplanned changes or cancellations in prenatal checkups can leave pregnant women feeling unprepared for childbirth, which can negatively impact their mental health and potentially lead to anxiety, stress, and both prenatal and postnatal depression [32]. Therefore, it is crucial to emphasize the importance of consistent prenatal checkups during infectious disease outbreaks to help pregnant women maintain their mental health.

This study also discovered that pregnant women reported increased levels of prenatal depression during the COVID-19 period when they encountered heightened pandemic-related pregnancy stress, diminished marital satisfaction, insufficient pregnancy healthcare practices, were part of a weekend couple, and were at a more advanced gestational age. These variables accounted for 38.2% of the variation in prenatal depression. Drawing on these findings, this study examined the influence of each factor on prenatal depression, proposed policy implications to tackle these issues, and suggested practical solutions for pregnant women.

Pandemic-related stress during pregnancy was identified as the most significant factor contributing to an increase in prenatal depression, a finding that aligns with previous research [6,33]. This specific type of stress, distinct from typical pregnancy stress, emerged as a major predictor of prenatal depression during the pandemic in this study. Notably, the incidence of prenatal depression was found to be twice as high during the pandemic as compared to pre-pandemic levels, suggesting that the pandemic itself has intensified depression symptoms. Moreover, both objective stressors, such as changes in prenatal checkups, financial difficulties, and unemployment, and subjective stressors, such as fear of COVID-19 infection and limited support during childbirth, have contributed to the heightened depression among pregnant women. In particular, higher levels of subjective stress were closely associated with more severe depression symptoms [6]. Additionally, variations in pandemic-related pregnancy stress were observed in relation to changes in childbirth plans due to the pandemic and pregnancies resulting from infertility treatments. This observation aligns with previous studies that employed similar methodologies [22,34]. Pregnant women infected with COVID-19 faced limited childbirth options, which escalated their fear and stress, potentially leading to PTSD [35]. Therefore, it is crucial to ensure that pregnant women have the right to make choices during childbirth in order to reduce stress during such crises. Women who became pregnant through infertility treatments experienced intense stress from the onset of their pregnancy. Concerns about treatment interruptions and delays during the pandemic [36], as well as the potential for decreased fertility due to infection [37], further exacerbated their depression and stress [36]. In response, policy discussions are needed to ensure the continuity of infertility treatments through medical insurance [36] and to incorporate prenatal care into emergency medical systems during pandemics. Previous research has shown that stress associated with childbirth and postpartum care significantly impacts prenatal depression during a pandemic [33]. Therefore, it is imperative to expand support and resources in prenatal care systems and to enhance pregnant women’s capabilities through prenatal education [33]. In addition, mindfulness interventions, particularly those delivered via mobile apps, have proven effective in reducing stress and alleviating prenatal depression during prolonged periods of infectious disease outbreaks. These interventions also provide high accessibility to mental health information and are well-accepted by pregnant women [38]. Therefore, developing digital health stress management programs that are readily available to pregnant women at any time and place, would be helpful in preparation for recurring infectious diseases.

In the current study, the second most influential factor on prenatal depression during the pandemic was identified as pregnancy healthcare practice behavior, aligning with previous research [10,39]. The extent of these behaviors appears to be influenced by factors such as marital status, family structure, marital satisfaction, self-quarantine experience during pregnancy, and income changes due to the COVID-19 pandemic. These factors are also linked to the social support provided by family members, suggesting that their emotional and material assistance significantly impacts how pregnant women manage their healthcare practices during the pandemic. Typically, pregnant women receive more social support from family and relatives than from friends. Adequate family support has been shown to positively influence health-promoting behaviors [40,41], a finding supported by prior research. This study also discovered that pandemic-related income changes affected pregnancy healthcare practice behaviors. This aligns with another study [40] that found insufficient income negatively impacts women’s health-promoting behaviors. Moreover, a household trend survey in Korea [42] confirmed that the pandemic has led to a decrease in income and an increase in unemployment, which in turn influences changes in household income. Therefore, identifying the material, emotional, and economic support available to pregnant women from family and friends during a pandemic situation and establishing measures to ensure that pregnant women can avoid deficiencies during self-quarantine and maintain their healthcare practices, can be helpful preventive measures against prenatal depression. In this context, promoting healthcare practices among pregnant women during the pandemic is of particular importance. For instance, providing virtual reality-based prenatal group exercise programs tailored to their altered lifestyles can positively impact their bonding with other pregnant women [43].

Prenatal depression was significantly influenced by marital satisfaction, which aligns with previous research [7]. Lower marital satisfaction, which often results in less support from husbands, has been reported as associated with an increase in prenatal depression [7]. This study also found that being a weekend couple, as opposed to living in a large or nuclear family, seemed to result in less support from husbands, which in turn influenced prenatal depression. However, a pre-COVID-19 study [7] found that prenatal depression was twice as prevalent in large families living with parents compared to nuclear families. This suggests the need for further research on family size and weekend couples, especially during pandemic situations. Given that an increase in domestic conflicts and violence were attributed to factors such as unemployment, school closures, and social isolation during the pandemic [44], such factors may likely influence pregnant women's marital satisfaction and should be considered for future research.

Finally, this study identified a correlation between advanced gestational age and prenatal depression, a finding that aligns with prior research [16]. All participants in this study were in their third trimester and exhibited an increase in prenatal depression as their gestational age progressed. Given that the third trimester is a crucial phase for the onset of prenatal depression, largely due to heightened physical and psychological stress [16], greater attention is required as pregnancy progresses, to facilitate early identification and efficient treatment of prenatal depression.

The current study revealed a relatively high level of pandemic-related pregnancy stress, and subscores of 2.03 for stress related to preparedness and 1.47 for perinatal infection. Using the same measurement, higher average scores were reported in prior studies: a US study [22] reported an average score of 3.36 for both subcategories, while an Italian study [45] reported scores of 2.75 and 2.59 for preparedness and perinatal infection stress, respectively. Interestingly, participants in our study experienced less pandemic-related pregnancy stress. This discrepancy may be due to the timing of the study. The US study [22] was conducted during a period of rapidly increasing COVID-19-related deaths [46], and the Italian study [45] took place during a second wave of the pandemic. In contrast, our study in Korea was carried out during a phase of relaxed social distancing measures [17] and COVID-19 transitioning to an endemic phase. This context may account for the lower stress levels observed among our Korean participants compared to those in the previous studies. This also suggests that as time passed, the level of stress experienced during the pandemic gradually decreased, indicating that people have been adapting to the new normal [47]. However, it is important to note that the various traumas experienced during the pandemic could potentially lead to depression or post-pandemic stress disorder even after the pandemic has ended [48]. Therefore, despite the lower levels of pandemic-related pregnancy stress observed among pregnant women in Korea, it is premature to be complacent, monitoring the trends of pregnancy stress as the pandemic concludes and in the subsequent periods would be beneficial.

The high level of pregnancy healthcare practice behaviors in this study (67.07 points) is comparable to the level reported in a pre-COVID-19 study in Korea [25], which recorded an average score of 63.47 to 65.32 using the same evaluation tool. Contrary to expectations that social distancing and isolation would decrease pregnancy healthcare practice behaviors, no such reduction was observed. Interestingly, pregnant women who did not experience social distancing exhibited higher healthcare practice behaviors than those who did. Moreover, women who received prenatal education during the COVID-19 pandemic demonstrated superior healthcare practices compared to those who did not. However, considering that only 33.9% of participants experienced social distancing and 53.3% received prenatal education during the pandemic, these factors did not significantly impact the overall level of the behaviors. Pregnant women who receive professional prenatal education, equipped with accurate prenatal knowledge, can enhance their self-care abilities and healthcare practice behaviors. While face-to-face education was previously the standard, recent advancements in digital technology and the proliferation of infectious diseases have led to the introduction of web- or mobile-based prenatal education programs [25,49]. In light of this, the aim of this study was to investigate the evolving needs and current status of prenatal education for pregnant women during the pandemic. Despite social distancing, self-quarantine, and public facility closures, 53.3% of participants had attended at least one prenatal education session. This is similar to the 53.7% reported in a pre-pandemic study [50]. Regardless of the outcome, a significant 87.2% of participants expressed a need for prenatal education, a figure that substantially exceeds the participation rate. The average interest in prenatal education was around 8 out of 10 points, indicating a significant surge in demand during this period. The primary reasons for not receiving prenatal education were “social distancing” and a “lack of information about when and where the education was available,” suggesting that pandemic-related restrictions were the main obstacles to receiving education.

The internet emerged as the primary source of prenatal information for pregnant women during the COVID-19 pandemic, accounting for 84.4% of all information sources, compared to 30.0% before the pandemic [51] and 82.4% just prior to the pandemic [52]. This indicates an increased dependence on the internet for information. During the pandemic’s peak in Korea, there was a significant decrease in the number of patients and visits to hospitals or clinics compared to the period before the outbreak. This led to a potential decrease in health services provided by primary healthcare facilities [53], and pregnant women may not have received sufficient prenatal information from health professionals, leading to a natural increase in their reliance on the internet. However, the reliability of internet information can be questionable [54], and the information available may not always cater to the specific needs of pregnant women [55]. Therefore, it is crucial to devise policy-level strategies to improve the digital health literacy of pregnant women. This will enable them to effectively search for, understand, and assess the reliability of online prenatal information [56]. Given the recurring nature of infectious diseases, it is imperative for clinical experts to focus on developing strategies that can positively influence pregnant women’s reliance on the internet for prenatal information.

This study also found that the level of prenatal depression was associated with the desired prenatal education mode. Pregnant women who favored face-to-face prenatal education exhibited higher instances of prenatal depression. This can be attributed to the fact that these women seek more than just information from their education; they also crave empathy and emotional support, which they find through bonding with other expectant mothers in similar circumstances [55]. However, online prenatal education may not provide the same opportunities for forming these emotional connections, potentially leading to feelings of isolation [57]. Before the pandemic, prenatal education in Korea was primarily conducted in person at public health centers. While online prenatal education can serve as an effective intervention for prenatal depression during a pandemic, it may not fully address the psychological and emotional needs that are met through social interactions. Consequently, further research is needed to develop effective online prenatal education programs that can be utilized during pandemic conditions.

Based on the findings of this study, the factors that significantly impacted prenatal depression included pandemic-related pregnancy stress, marital satisfaction (or lack thereof), pregnancy healthcare practices, family type (specifically, weekend couples), and gestational age. However, as this study focused solely on women in their third trimester, the results may not be directly applicable to those in their first or second trimesters. Additionally, the survey used to assess prenatal education was conducted in a straightforward question-and-answer format, which limited the ability to provide a comprehensive overview of prenatal education practices during the pandemic. The factors associated with prenatal depression also had a relatively low explanatory power of 38.2%. This could be due to the fact that unlike previous research conducted in Korea during the pandemic [16], this study did not specifically analyze pregnant women with a history of depression or those currently experiencing depression during pregnancy. Despite these limitations, the study’s significance lies in its examination of the changing phenomena by analyzing each variable of pandemic-related pregnancy stress and pregnancy healthcare practices in relation to the characteristics of pregnant women and their prenatal education. The study also provides foundational data for the development of various prenatal education programs aimed at promoting mental health in pregnant women in preparation for future infectious diseases. It further underscores the need for strategies to reduce pregnancy stress and improve pregnancy healthcare behaviors.

In conclusion, prenatal depression among pregnant women during pandemics like COVID-19 is a serious issue that necessitates immediate evaluation and treatment. Because prenatal depression often intensifies in the later stages of pregnancy, interventions that are both timely and tailored to the pregnancy stage are essential. It is critical to acknowledge stress and healthcare practice behaviors as significant influences on prenatal depression during the COVID-19 pandemic. Therefore, monitoring and managing these factors among pregnant women is crucial, particularly in the face of recurring infectious diseases. Consequently, national and healthcare policies, as well as active interventions, are required to address these issues.

## Figures and Tables

**Table 1.** General, obstetric, and prenatal education characteristics of the participants (N=180)

Characteristics	Categories	n (%)
*General characteristics*		
Age (year)	Mean±SD 32.22±2.86 (Min 26, Max 41)	
	18–29	27 (15.0)
	30-34	119 (66.1)
	≥35	34 (18.9)
Marital status	Married	176 (97.8)
	Common-law marriage	4 (2.2)
Family type	Couple with child(ren)	164 (91.1)
	Couple with parent(s)	9 (5.0)
	Weekend couple	7 (3.9)
Marital satisfaction	Satisfied	165 (91.7)
	Moderate	11 (6.1)
	Dissatisfied	4 (2.2)
Job loss due to the COVID-19 pandemic	Yes	24 (13.3)
	No	156 (86.7)
Changes in income due to the COVID-19 pandemic	No	127 (70.6)
	Yes	53 (29.4)
*Obstetric characteristics*		
Gestational age (week)	Median 31 weeks+6 days	
	28-31+6 days	112 (62.2)
	32-35+6 days	39 (21.7)
	≥36	29 (16.1)
Prenatal checkup	Regular	162 (90.0)
	Irregular	18 (10.0)
Parity	Nullipara	159 (88.3)
	Multipara	21 (11.7)
Planned pregnancy	Yes	144 (80.0)
	No	36 (20.0)
Smoking during pregnancy	Yes	8 (4.4)
	No	172 (95.6)
Drinking during pregnancy	Yes	11 (6.1)
	No	169 (93.9)
Pregnancy method	Natural	174(96.7)
	Infertility treatment	6(3.3)
High-risk pregnancy	Yes	21 (11.7)
	No	159 (88.3)
Desired sex of fetus	Yes	144 (80.0)
	No	36 (20.0)
Preferred childbirth method	Natural delivery	136 (75.6)
	Cesarean section	42 (23.3)
	Undecided	2 (1.1)
Change in childbirth plan due to the COVID-19 pandemic	Yes	24 (13.3)
	No	156 (86.7)
Experience of self-quarantine during pregnancy	Yes	61 (33.9)
	No	119 (66.1)
Changes in prenatal checkups due to the COVID-19 pandemic	Yes	46 (25.6)
	No	134 (74.4)
COVID-19 diagnosis during pregnancy	Yes	27 (15.0)
	No	153 (85.0)
*Characteristics of prenatal education*		
Need for prenatal education	Much needed	102 (56.7)
	Needed	55 (30.5)
	Somewhat needed	23 (12.8)
Interest in prenatal education	Mean±SD 7.90±1.79 (Min 2, Max 10)	
How prenatal information was obtained during pregnancy^†^	Internet (blogs, online communities, YouTube)	152 (84.4)
	Healthcare providers (nurses, doctors, midwives)	93 (51.7)
	Acquaintances	63 (35.0)
	Prenatal classes at hospitals, public health centers, etc. (including online)	58 (32.2)
	Books	53 (29.4)
Desired prenatal education method	Knowledge transfer education	20 (11.1)
	Knowledge transfer education and practice	140 (77.8)
	Practice education	20 (11.1)
Desired prenatal education mode	Digital/electronic media (online)	98 (54.4)
	Face-to-face	72 (40.0)
	Virtual reality, augmented reality	10 (5.6)
Changes in prenatal education participation due to the COVID-19 pandemic	Yes	46 (25.6)
	Reasons for changes in prenatal education^†^	
	Schedule change or cancellation of classes by institutions offering prenatal education	29 (63.0)
	Cancelled due to fear of COVID-19 infection	24 (52.2)
	Self-quarantine due to COVID-19 infection	16 (34.8)
	No	63 (35.0)
	Did not apply for prenatal education	71 (39.4)
Received prenatal education during the COVID-19 pandemic	Yes	96 (53.3)
	Satisfaction with the prenatal education Mean±SD 8.39±1.34 (Min 2, Max10)	
	No	
		84 (46.7)
	Reasons for not receiving prenatal education^†^	
	Restrictions on gatherings due to social distancing	45 (53.6)
	Did not know when and where classes on prenatal education would be held	39 (46.4)
	Schedule constraints	22 (26.2)
	Not interested	17 (20.2)
	Lack of equipment or difficulty accessing it online	16 (19.1)
	Thought it would not be helpful	9 (10.7)
Number of prenatal education classes attended	Mean±SD 3.33±2.76 (Min 1, Max 12)	
Accompanied by husband (or a guardian)	Yes	62 (64.6)
	No	34 (35.4)

COVID-19, coronavirus disease 2019.

† Multiple responses

**Table 2.** Pandemic-related pregnancy stress, pregnancy healthcare practice behavior, and prenatal depression (N=180)

Variable	Categories	Possible score range	Data range	Mean±SD (point average^†^)
Pandemic-related pregnancy stress	Total	7–35	7–35	24.50±6.37 (3.50±0.91)
	Preparedness stress	4–20	4–20	14.22±3.88 (2.03±0.55)
	Perinatal infection stress	3–15	3–15	10.29±3.09 (1.47±0.40)
Pregnancy healthcare practice behavior	Total	17–85	38–85	67.07±9.20 (3.95±0.54)
Prenatal depression		0–30	0–27	8.85±5.31
	No (0–9) (n=91, 50.6%)		0–9	4.51±3.12
	Yes (≥10) (n=89, 49.4%)		10–27	13.28±2.86

† On a scale of 1-5 points.

**Table 3.** Differences in pandemic-related pregnancy stress, pregnancy healthcare practice behavior, and prenatal depression according to the participants’ general, obstetric, and prenatal education characteristics (N=180)

Characteristics	Categories	Pandemic-related pregnancy stress		Pregnancy healthcare practice behavior		Prenatal depression	
		Mean±SD	t/F/Z/χ^2^ (*p*)	Mean±SD	t/F/Z/χ^2^ (*p*)	Mean±SD	t/F/Z/χ^2^ (*p*)
*General characteristics*							
Age (year)^†^	18-29	23.07±4.38	3.73 (.155)	65.37±9.27	2.13 (.345)	10.18±6.13	4.78 (.092)
	30-34	24.76±6.55		67.74±8.41		8.18±4.99	
	≥35	24.76±7.04		66.06±11.57		10.12±5.46	
Marital status^‡^	Married	24.57±6.39	–1.18 (.236)	67.47±8.84	–3.07 (.002)	8.71±5.17	–1.44 (.151)
	Common-law marriage	21.50±5.32		49.25±8.06		14.75±8.81	
Family type^†^	Couple+child(ren)	24.44±6.54	0.13 (.939)	66.55±9.12	7.17 (.028)	8.95±5.22	11.84 (.003)
	Couple+parent(s)	25.78±1.71		75.00±7.74		3.44±3.78	
	Weekend couple	24.28±6.63		68.86±9.01		13.43±3.91	
Marital satisfaction^†^	Satisfied	24.73±6.43	5.73 (.057)	68.05±8.60	18.94 (<.001)	8.34±5.02	11.79 (.003)
	Moderate	20.82±4.24		55.64±10.03		12.18±3.63	
	Dissatisfied	25.50±6.81		58.00±5.60		20.50±5.07	
Job loss due to the COVID-19 pandemic^‡^	Yes	26.33±5.78	–1.53 (.126)	64.96±10.08	–1.47 (.142)	10.83±5.052	–2.21 (.027)
	No	24.22±6.43		67.39±9.05		8.54±5.30	
Changes in income due to the COVID-19 pandemic	No	24.32±6.27	–0.59 (.553)	68.00±8.95	2.13 (.035)	8.01±5.00	–3.35 (.001)
	Yes	24.94±6.64		64.83±9.51		10.85±5.55	
*Obstetric characteristics*							
Gestational age^†^	28-31+6 days	24.87±6.59	1.65 (.439)	67.68±8.65	5.40 (.067)	8.24±5.77	4.18 (.123)
	32-35+6 days	24.05±5.08		68.31±9.01		10.08±4.22	
	≥36	23.69±7.13		63.03±10.69		9.55±4.48	
Prenatal checkup^‡^	Regular	24.72±6.47	–1.73 (.084)	68.21±8.47	–4.28 (<.001)	8.52±5.35	–2.73 (.006)
	Irregular	22.61±5.11		56.78±9.40		11.83±3.91	
Parity^‡^	Nullipara	24.36±6.35	–0.75 (.453)	67.40±8.99	–1.59 (.111)	8.72±5.19	–1.19 (.234)
	Multipara	25.57±6.57		64.52±10.60		9.86±6.22	
Planned pregnancy	Yes	24.67±6.35	0.68 (.499)	68.05±8.79	2.94 (.004)	8.41±4.99	–2.25 (.026)
	No	23.86±6.51		63.11±9.87		10.61±6.20	
Smoking during pregnancy^‡^	Yes	28.37±5.83	–1.88 (.060)	57.62±10.13	–2.62 (.009)	11.00±4.00	–1.32 (.186)
	No	24.32±6.35		67.50±8.95		8.75±5.35	
Drinking during pregnancy^‡^	Yes	25.64±7.14	–0.65 (.518)	56.18±9.73	–3.54 (<.001)	12.27±3.63	–2.48 (.013)
	No	24.43±6.33		67.77±8.74		8.63±5.34	
Pregnancy method^‡^	Natural	24.36±6.40	–2.01 (.045)	67.19±9.21	–1.21 (.227)	8.86±5.32	–0.20 (.839)
	Infertility treatment	28.67±3.93		63.33±8.80		8.50±5.54	
High-risk pregnancy^‡^	Yes	26.38±7.05	–1.70 (.089)	61.67±11.57	–2.46 (.014)	10.81±4.86	–1.73 (.084)
	No	24.26±6.26		67.78±8.64		8.59±5.33	
Desired sex of fetus	Yes	24.58±6.25	0.30 (.766)	67.10±9.03	0.11 (.913)	8.35±5.12	–2.54 (.012)
	No	24.22±6.94		66.92±10.00		10.83±5.67	
Preferred childbirth method^†^	Normal delivery	24.80±6.31	1.49 (.475)	67.95±8.82	3.82 (.148)	8.39±5.47	6.37 (.041)
	Cesarean section	23.40±6.63		64.36±10.03		10.43±4.61	
	Undecided	27.50±0.71		63.50±9.19		7.00±1.41	
Change in childbirth plan due to the COVID-19 pandemic^‡^	Yes	28.58±4.35	–3.62 (<.001)	66.46±10.26	–0.45 (.652)	9.33±4.64	–0.56 (.573)
	No	23.88±6.41		67.16±9.06		8.77±5.42	
Experience of self-quarantine during pregnancy	Yes	25.33±5.54	1.24 (.216)	64.62±9.54	–2.59 (.010)	10.61±4.93	3.26 (.001)
	No	24.08±6.74		68.32±8.80		7.95±5.30	
Changes in prenatal checkups due to the COVID-19 pandemic	Yes	25.93±6.16	1.77 (.078)	66.43±8.05	–0.54 (.591)	10.37±4.88	2.27 (.024)
	No	24.01±6.39		67.28±9.59		8.33±5.37	
COVID-19 diagnosis during pregnancy^‡^	Yes	25.55±5.34	–0.65 (.517)	64.89±11.18	–1.20 (.231)	10.30±4.90	–1.65 (.100)
	No	24.32±6.53		67.45±8.80		8.59±5.36	
*Characteristics of prenatal education*							
Need for prenatal education^†^	Much needed	25.68±7.05	22.51 (<.001)	68.19±9.14	5.06 (.080)	8.70±5.58	0.87 (.646)
	Needed	24.09±5.13		66.69±8.38		9.31±4.88	
	Somewhat needed	20.30±3.51		63.00±10.47		8.39±5.24	
Desired prenatal education method^†^	Knowledge transfer education	26.55±7.29	9.41 (.009)	72.25±7.62	16.38 (<.001)	10.40±5.02	3.73 (.155)
	Knowledge transfer education	24.68±6.11		67.14±9.22		8.49±5.36	
	and practice						
	Practice education	21.25±6.38		61.35±7.43		9.80±5.12	
Desired prenatal education mode^†^	Digital/electronic media (online)	24.19±6.86	0.621 (.733)	69.19±8.94	13.21 (.001)	7.85±5.32	6.79 (.034)
	Face-to-face	24.72±5.83		64.17±9.01		10.26±5.21	
	VR, AR	26.00±5.35		67.10±8.14		8.50±3.81	
Changes in prenatal education participation due to the COVID-19 pandemic	Yes^a^	26.59±5.75	5.54 (.005)	65.78±9.58	3.41 (.035)	9.89±5.51	1.24 (.291)
	No^b^	24.95±7.52	a>c	69.48±7.80	a, c<b	8.65±4.90	
	Did not apply for	22.76±5.14		65.76±9.78		8.35±5.51	
	prenatal education^c^						
Receipt of prenatal education during the COVID-19 pandemic	Yes	26.01±6.85	3.49 (.001)	68.75±8.47	2.67 (.008)	8.85±5.42	0.01 (.991)
	No	22.78±5.30		65.14±9.67		8.84±5.22	

AR, augmented reality; COVID-19, coronavirus disease 2019; VR, virtual reality.

† Kruskal-Wallis test,

‡ Mann-Whitney U-test.

**Table 4.** Correlation between the participants’ general and obstetric characteristics, characteristics of prenatal education, pandemic-related pregnancy stress, pregnancy healthcare practice behavior, and prenatal depression during the pandemic (N=180)

Variable	r (*p*)			
	Age	Gestational age	Pandemic-related pregnancy stress	Pregnancy healthcare practice behavior
Age	1			
Gestational age	.09 (.212)	1		
Pandemic-related pregnancy stress	.03 (.732)	–.07 (.339)	1	
Pregnancy healthcare practice behavior	–.04 (.588)	–.21 (.006)	.01 (0.947)	1
Prenatal depression	.03 (.699)	.18 (.019)	.27 (<.001)	–.42 (<.001)

**Table 5.** Factors affecting prenatal depression (N=180)

Variable	B	SE	β	T (*p*)	VIF
(Constant)	1.48	5.29		0.28 (.780)	
Family type^†^					
Couple+child(ren)	2.65	1.51	0.14	1.75 (.081)	1.91
Weekend couple	6.26	2.21	0.23	2.84 (.005)	1.88
Marital satisfaction^†^					
Moderate	2.02	1.51	0.09	1.33 (.185)	1.35
Dissatisfied	9.24	2.52	0.26	3.66 (*<*.001)	1.42
Job loss due to the COVID-19 pandemic (yes)^†^	–0.35	1.15	–0.02	–0.30 (.763)	1.56
Changes in income due to the COVID-19 pandemic (yes)^†^	1.02	0.84	0.09	1.21 (.227)	1.52
Prenatal checkup (irregular)^†^	1.12	1.23	0.06	0.91 (.364)	1.41
Planned pregnancy (no)^†^	–0.60	0.88	–0.05	–0.68 (.498)	1.27
Drinking during pregnancy (yes)^†^	–0.16	1.51	–0.01	–0.11 (.915)	1.34
Desired sex of fetus (no)^†^	1.25	0.85	0.10	1.46 (.145)	1.20
Preferred childbirth method^†^ (normal delivery)	–1.23	0.77	–0.10	–1.59 (.114)	1.14
Experience of self-quarantine during pregnancy (yes)^†^	0.53	0.74	0.05	0.71 (.477)	1.28
Changes in prenatal checkups due to the COVID-19 pandemic (yes)^†^	1.13	0.83	0.09	1.37 (.174)	1.35
Desired prenatal education mode^†^					
Face-to-face	0.37	0.70	0.03	0.52 (.605)	1.22
VR, AR	0.33	1.44	0.01	0.23 (.818)	1.12
Gestational age	0.04	0.02	0.15	2.32 (.022)	1.25
Pandemic-related pregnancy stress	0.24	0.05	0.29	4.70 (*<*.001)	1.10
Pregnancy healthcare practice behavior	–0.14	0.04	–0.25	–3.31 (.001)	1.60
R^2^ =.444, adjusted R^2^=.382, F=7.14, *p*<.001					

AR, augmented reality; B, unstandardized coefficients; β, standardized coefficients; VIF, variance inflation factor; VR, virtual reality.

† References: family type (couple+parent[s]); marital satisfaction (satisfied); job loss due to the COVID-19 pandemic (no); changes in income due to the COVID-19 pandemic (no); prenatal checkup (regular); planned pregnancy (yes); drinking during pregnancy (no); desired sex of fetus (yes); preferred childbirth method (undecided); experience of self-quarantine during pregnancy (no); changes in prenatal checkup due to the COVID-19 pandemic (no); desired prenatal education mode (digital/electronic media [online]).
